# Proposal and Phylogenetic Relationships of *Lapsamita*, a New Genus of Lapsiines, and Description of a New Species (Araneae, Salticidae)

**DOI:** 10.1371/journal.pone.0056188

**Published:** 2013-02-28

**Authors:** Gustavo R. S. Ruiz

**Affiliations:** Instituto de Ciências Biológicas, Universidade Federal do Pará, Belém, Pará, Brazil; California State University Fullerton, United States of America

## Abstract

*Lapsamita maddisoni* gen. et sp. nov. is described from Bahia, Brazil. The presence of palpal claws in females, long ventral spines on front tibiae and metatarsi and long retrolateral tibial apophyses in male palps suggested that the species was closely related to or even nested within *Soesiladeepakius* Makhan, 2007. To evaluate its phylogenetic position, a cladistic analysis was carried out using a data matrix comprising 24 morphological characters, scored for 13 taxa. The analysis showed *L. maddisoni* as the sister group of *Soesiladeepakius*, supporting the proposal of the new genus. Character evolution is discussed and compared to a previous study on the group.

## Introduction

The few groups of jumping spiders not included in the large clade Salticoida [1] have been the focus of several phylogenetic studies [1]–[6]. Aims of those studies include revealing the early evolution of the family and providing better outgroup choices for phylogenetic studies on major spider clades.

Among the non-salticoid groups, the lyssomanines (*sensu stricto*) and the lapsiines have flourished in the Neotropics. Despite their cryptic green bodies and their habit of sitting on large green leaves, lyssomanines are relatively conspicuous and easily collected by untrained arachnologists using beating sheets. Lapsiines, however, apparently show narrower microhabitat preferences and are difficult to find, which has hindered knowledge of their biodiversity.

Lapsiines, yet to be formalized as a subfamily, so far includes *Lapsias* Simon, 1900 and three recently discovered genera, *Galianora* Maddison, 2006, *Thrandina* Maddison, 2006 and *Soesiladeepakius* Makhan, 2007. *Galianora* and *Thrandina* were immediately recognized as non-salticoids [4], but the non-salticoid condition of *Soesiladeepakius* was corroborated later using DNA sequences [6]. *Soesiladeepakius* was erroneously thought [7] to be a thiodinine (Salticoida: Amycoida). Several synapomorphies would support the monophyly of *Soesiladeepakius* [6], such as abdominal clusters of white scales, elongate ventral spines on front legs, an anterior pocket on the epigynal plate formed by a sclerotized U-shaped rim and the loss of the flexible median apophysis in the male palp, among others.

Despite the vast territory, the only lapsiine genus recorded to date from eastern South America is *Soesiladeepakius*, apparently restricted to the Amazonian region, but examination of material recently collected from areas of Atlantic rainforest in the state of Bahia, Northeastern Brazil, resulted in the recognition of an undescribed species with palpal claws in females. This species also had some of those characters mentioned as synapomorphies for *Soesiladeepakius*, namely the abdominal white scales, long ventral spines on front legs, the anterior epigynal pocket and no flexible median apophyses, as well as the long retrolateral tibial apophyses, seen in some species of *Soesiladeepakius*. Assuming the common characters were synapomorphic, this undescribed species could represent a lineage closely related to *Soesiladeepakius*, or even be nested within the genus, possibly related to the long-RTA species such as *S. lyra* Ruiz & Maddison, 2012. The new species is herein described and a phylogenetic analysis to evaluate its position is given.

## Materials and Methods

Specimens are deposited in the Museu Emílio Goeldi, Belém (MPEG). Measurements are in millimeters. Total length includes anterior median eyes and anal tubercle [8]. Spine description follows the standard for spiders [9]. Abbreviation used throughout the text are: (d) dorsal, (di) distal, (p) prolateral, (r) retrolateral, (RFA) retrolateral femoral apophysis, (RTA) retrolateral tibial apophysis, (v) ventral.

In order to verify the phylogenetic position of the new species, I carried out an analysis using morphological characters. The new taxon was added to the morphological matrix for lapsiines given in a previous study [6], and the 24 characters of that matrix were scored for this species. That matrix included all the known lapsiine genera, all the known species of *Soesiladeepakius* and *Portia africana* (Simon), a spartaeine (a related Old World non-salticoid), to root the tree. The matrix was edited in Mesquite [10] and non-applicable and unknown data entered the matrix as ‘–’ and ‘?’, respectively. Using parsimony, characters were equally weighted and multistate characters were coded as non-additive [11], [12]. Searches for the most parsimonious trees were carried out in TNT, ver. 1.1 [13], including 20,000 random addseqs, ratchet (100 iterations), drift (100 cycles) and trees were collapsed after search. Trees obtained were compared and character optimization was read directly from the cladogram using WinClada, ver. 1.00.08 [14].

### Nomenclatural acts

The electronic edition of this article conforms to the requirements of the amended International Code of Zoological Nomenclature, and hence the new names contained herein are available under that Code from the electronic edition of this article. This published work and the nomenclatural acts it contains have been registered in ZooBank, the online registration system for the ICZN. The ZooBank LSIDs (Life Science Identifiers) can be resolved and the associated information viewed through any standard web browser by appending the LSID to the prefix “http://zoobank.org/”. The LSID for this publication is: urn:lsid:zoobank.org:pub:31560D20-5235-4E3E-BB0B-C486AF2FBD42. The electronic edition of this work was published in a journal with an ISSN, and has been archived and is available from the following digital repositories: PubMed Central, LOCKSS.

***Lapsamita*** **new genus** (urn:lsid:zoobank.org:act:88A38185-662F-4C77-8480-13534024EDD4)

#### Etymology

The name is a combination of *Lapsias* Simon, the first genus of lapsiines described, and the Latin noun *amita* ( = aunt). Gender is feminine.

#### Diagnosis

**Figure 1:**
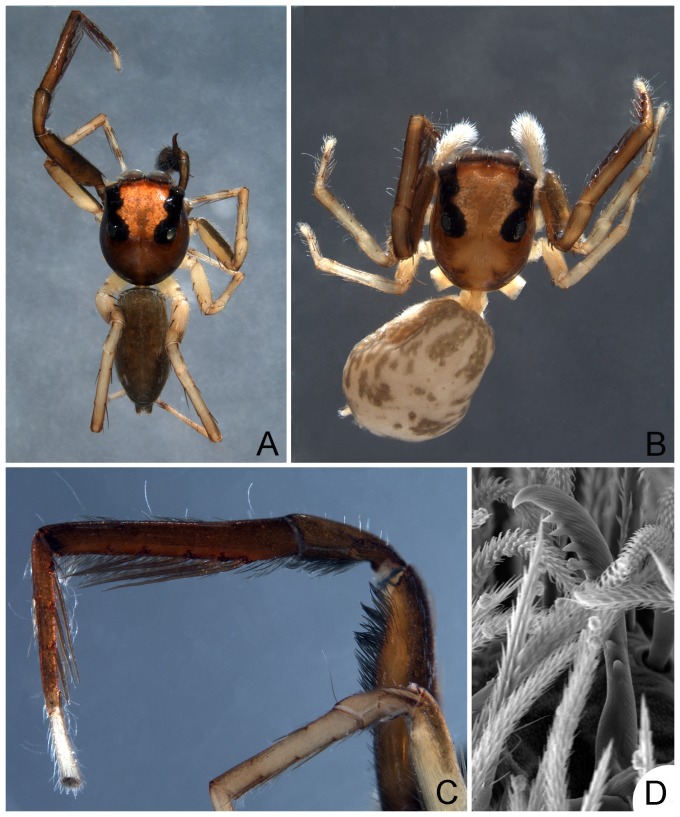
*Lapsamita maddisoni* gen. et sp. nov. - A. holotype male, dorsal view. - B. paratype female, dorsal view. - C. male left leg I, retrolateral view. - D. female palpal claw, ventral view.

Among the neotropical non-salticoids, recognized by the palpal claw in females, members of *Lapsamita* can be distinguished by the presence of 5 pairs of long ventral spines on front tibiae and 4 ventral pairs on metatarsi (Fig. 1C).

#### Description

**Figure 2:**
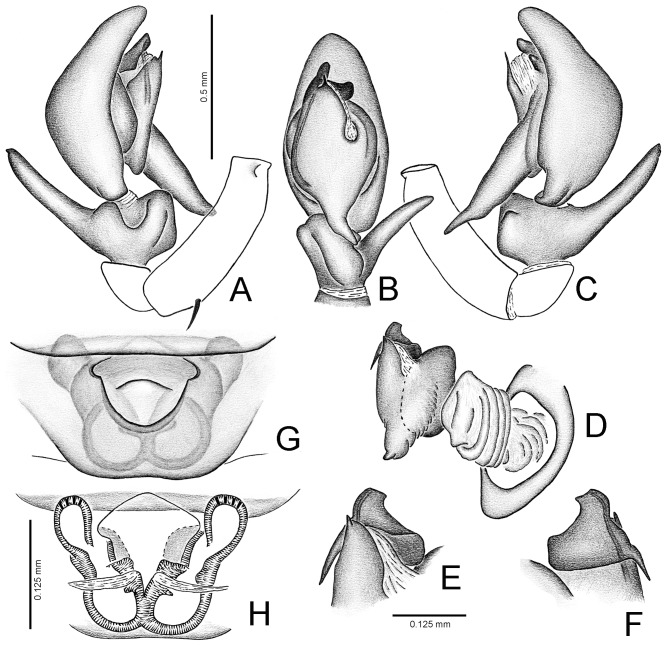
*Lapsamita maddisoni* gen. et sp. nov. - A. left male palp, prolateral view. - B. same, ventral view. - C. same, retrolateral view. - D. distal portion of left male palp, expanded, bulb in retroventral view. - E. detail of expanded embolus, retroventral view. - F. same, dorsal view. - G. female epigyne, ventral view. - H. same, dorsal view, cleared.

Medium-sized yellowish jumping spiders with darker carapaces and front legs (Figs. 1A–B). Carapace elliptical. Salticoid eye pattern. Black rings around eyes very conspicuous. Chelicera small and vertical in both sexes, with 3 promarginal and 2 retromarginal teeth (typical lapsiine dentition). Palpal femur with no depressions or apophyses; RTA very long, relocated dorsally. Tegulum globular with a long posterior projection (Fig. 2C). No articulated median apophysis (Fig. 2B). Embolus short, flexible, with a large base and an acute tegular apophysis (conductor?) adjacent to it (Fig. 2E). Females with palpal claw (Fig. 1D). Strong ventral spines on leg I in both sexes (Fig. 1C). Other leg spines of normal size. Neither sex with dorsal abdominal scutum (Figs. 1A–B). Epigyne with anterior transverse flap, small openings on the inner sides of a large atrium and short copulatory ducts converging towards the posterior border, where small spermathecae are located (Figs. 2G–H); large globose glandular (nutritive?) portions associated with the copulatory openings (Fig. 2H).

***Lapsamita maddisoni*** **sp. nov.** (urn:lsid:zoobank.org:act:6EB407C6-C268-43E1-AE40-843B2D9ABEA1)

Figs. 1A–D, 2A–H

#### Type

Holotype: male from Serra da Jiboia, Santa Teresinha, Bahia, Brazil (Estrada Pioneira, near the antenna, coordinates -12.870833, -39.481111), 08.XI.2010, L.S. Carvalho, deposited in MPEG 19193. Paratype: 2 females, same data (MPEG 19194).

#### Etymology

The specific epithet is in honor of the distinguished Canadian arachnologist Dr. Wayne P. Maddison, for his pioneering research on salticid systematics.

#### Diagnosis

See generic diagnosis.

#### Description

Holotype male. Total length: 3.37. Carapace brown, 1.70 long, 1.32 wide, 0.95 high, with conspicuous light intestinal diverticuli seen through the tegument among the eyes (Fig. 1A). Ocular quadrangle 1.00 long. Anterior eye row 1.20 wide, posterior 1.05 wide. Clypeus low, not covered by modified scales. Chelicera brown. Endite rounded, light brown. Labium light brown. Sternum yellow. Palp dark brown. No RFA. Palpal tibia with a square ventral projection, a small prolateral projection and a very long RTA, relocated retrodorsally, reaching almost half the length of the cymbium (Figs. 2A, 2C). No gland inside RTA. Cymbium ventrally curved. Tegulum rounded with a posterior acute projection, with no median apophysis; a small, acute projection at the distal end of the tegulum accompanying the embolus (Figs. 2B, 2E); sperm duct with no loop before entering the embolus; embolus short, articulated by an embolic hematodocha, with a large base bearing a prolateral projection pointing posteriorly (Figs. 2D–F). Legs 1432. Length of femur I: 1.90, II: 1.00, III: 1.00, IV: 1.40, patella + tibia I: 2.00, II: 1.25, III: 1.12, IV: 1.60, metatarsus + tarsus I: 1.40, II: 1.20, III: 1.40, IV: 2.15. Leg spines: femur I d1-1-1, p1di, II–IV d1-1-1, p1di, r1di; patella I p1, r1, II p1, r0, III–IV p1, r1; tibia I p1-1, r1-1, v2-2-2-2-2 (elongate, Fig. 1C), II p1-1, r1-1, v2-2-2, III p1-1, r1-1, v2di, IV p1-1, r1-1, v1p-2; metatarsus I v2-2-2-2 (elongate, Fig. 1C), II p2-1, r2-1, v2-2, III–IV p1-2, r1-2, v1-1. Leg I brown, with yellow tarsus and a fringe of dark hairs along the retroventral keel of femur (Fig. 1C). Leg II yellow, with a longitudinal dark brown band on the ventroprolateral face of femur (Fig. 1A). Legs III–IV yellow. Abdomen dorsally brown, not covered by conspicuous scutum, with at least a pair of tufts of white scales at the middle of its length and a second pair on the posterior half (Fig. 1A); ventrally with a wide, dark brown, longitudinal stripe. Spinnerets dark brown.

*Paratype female.* Total length: 3.25. Carapace 1.50 long, 1.25 wide, 0.80 high. Ocular quadrangle 0.90 long. Anterior eye row 1.15 wide, posterior 1.00 wide. Chelicera, endite, labium and sternum as in male. Legs 4123. Length of femur I: 1.10, II: 0.95, III: 0.85, IV: 1.25, patella + tibia I: 1.65, II: 1.10, III: 0.95, IV: 1.50, metatarsus + tarsus I: 1.15, II: 1.05, III: 1.20, IV: 1.40. Leg spines: femur I–IV d1-1-1, p1di, r1di; patella I–IV p1, r1; tibia I p1-1, r0-1, v2-2-2-2-2 (elongate), II p1-1, r1-1, v2-2-2, III p1-0-1, r1-1-1, v2di, IV p1-1, r1-1 (or r1-0-1-1), d0 (or d1di), v1p-2 (or v1p-3); metatarsus I v2-2-2-2 (elongate), II p2-2, r1-2, v2-1r-2, III p2-2, r1-2, v1-1, IV p1-2, r1-2, v1-1. General coloration as in male, except for the following: palp white (Fig. 1B); abdomen cream colored with dorsal pattern (Fig. 1B); spinnerets yellow. The general coloration in the female is lighter than that in the male, for which reason is clearly visible a longitudinal dark brown band on the ventroprolateral face of femur I (Fig. 1B, similar to that present on leg II), as well as a second dark brown band, along the dorsoretrolateral face of the same article. Epigyne with an anterior transverse flap, a large atrium defined by a sclerotized U-shaped rim ( = anterior pocket), small openings on its inner sides and short copulatory ducts converging towards the posterior border, where small spermathecae are located (Figs. 2G–H); large globose glandular (nutritive?) portions associated with the copulatory openings (Fig. 2H).

### Phylogenetic relationships

*Lapsamita maddisoni* sp. nov. was added to the morphological matrix for lapsiines given in a previous study [6] and the 24 characters of that matrix were scored for this species, as follows: 0 1 0 0 1 ? 1 0 0 0 0 0 1 0 0 ? 1 1 0 1 ? 0 1 0. The cladistic analysis resulted in two equally parsimonious trees of 32 steps (Consistency Index  =  78, Retention Index  =  90). In both trees, *L. maddisoni* was recovered as the sister group of *Soesiladeepakius*. Except for the presence of *L. maddisoni*, the relationships among the groups were identical as those previously found [6], and so was the difference between the two trees. In one of them, *Galianora* showed up as the sister group of *Lapsamita* + *Soesiladeepakius*, and in the other, *Galianora* formed a polytomy with *Thrandina* and *Lapsias*, established by the presence of the flexible median apophysis in the male palp.

## Discussion

Our tree root, *Portia africana*, is a spartaeine known for having lost the median apophysis. Since the presence of a flexible median apophysis in the male palp is a plesiomorphy for salticids, the tree with the *Galianora*-*Thrandina*-*Lapsias* polytomy is rejected, because it assumes the change absent-to-present as the synapomorphy of this clade. The inclusion of other spartaeines in the analysis, such as *Holcolaetis* Simon or *Sonoita* Peckham & Peckham, who do have flexible median apophyses, could corroborate this decision.

**Figure 3:**
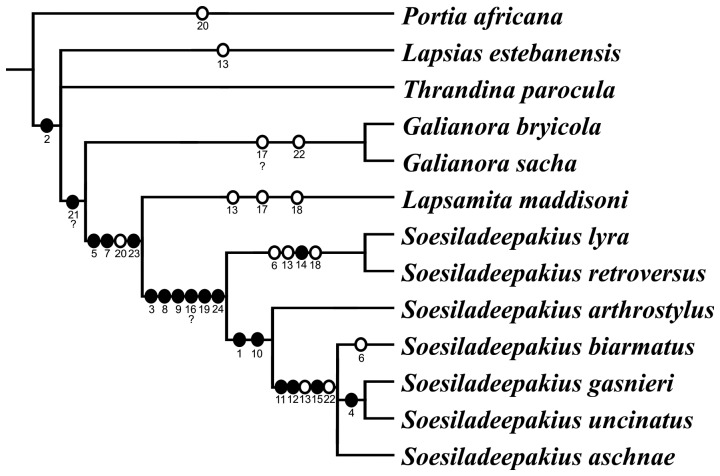
Chosen best tree of phylogenetic reconstruction of the lapsiines using morphological data. White and black circles on branches represent, respectively, homoplasious and non-homoplasious transformations. Numbers under circles indicate characters; for detailed character description and discussion, including question marks under numbers, see previous study [6].

In the other shortest tree, *Galianora* showed up as the sister group of *Lapsamita* + *Soesiladeepakius*. Although we still do not have molecular data available for *Lapsamita maddisoni*, the closer relationship between *Galianora* and *Soesiladeepakius* was found in analyses using DNA sequences [6], and the topology (*Galianora* (*Lapsamita* + *Soesiladeepakius*)) is expected to be corroborated in forthcoming molecular studies. This topology is chosen in this paper to discuss character evolution (Fig. 3).

Of all the 24 characters scored for the 13 taxa, the great majority exhibited the same optimization presented in the previous study [6] and are not discussed here. Nevertheless, some changes found in that study as synapomorphies for *Soesiladeepakius* appeared in this new analysis as synapomorphies joining that genus and *L. maddisoni*. Among these are: (a) conspicuous dorsal clusters of white scales on male abdomen (state 1, present; char. 5), (b) ventral spines on front legs (state 1, elongate, with all tips close to each other; char. 7), (c) median apophysis flexibly attached to tegulum at non sclerotized area (state 1, absent; char. 20) and (d) anterior epigynal pocket formed by a sclerotized U-shaped rim (state 1, present; char. 23). Because of that, synapomorphies of *Soesiladeepakius*, in this new data set, would be reduced to the male abdominal scutum (char. 3), fringe of white scales on dorsal male palpal femur (char. 8), male palpal femur with retroventral keel (char. 9), a proximal tegular apophysis (char. 19) and lateral ducts running anteriorly in the epigyne, possibly leading to copulatory openings (char. 24) (Fig. 3).

Other changes found in the previous study as non-homoplastic using that data set [6] showed up as homoplastic in this new analysis. The prolateral loop of the sperm duct (towards median apophysis, just before entering the embolus; char. 17), previously lost only in *Galianora bryicola* (to be confirmed in *G. sacha*), in this analysis was found as having homoplastically been lost in *L. maddisoni*. Despite being ambiguous, the delayed transformation (DELTRAN) algorithm is preferred because the repeated loss of that loop, if presence is confirmed to be plesiomorphic for the family, is already documented in the literature for many salticid groups and the accelerated transformation (ACCTRAN) would render the exact same loop of the sperm duct to reverse into present in *Soesiladeepakius*. The same case is applied to the conical shape of proximal tegulum (char. 18), previously found as a synapomorphy of *S. lyra* + *S. retroversus*, now homoplastic in *L. maddisoni*. Also ambiguous, the DELTRAN algorithm is preferred, because there seems to be no trace of the projected proximal tegulum in the other species of *Soesiladeepakius* and the cone is ventrally projected in *L. maddisoni* (dorsally in the other two species), suggesting the convergence.

*Lapsamita maddisoni* clearly presents a complete loss of the median apophysis and its discovery does not bring further clarification onto the evolution of this structure within laspiines. So far, median apophyses are present as typical sclerites articulating with the tegulum in *Lapsias*, *Thandina* and *Galianora*, but could be fused, lacking articulation, in *Soesiladeepakius*. Instead of not being articulated, these structures could also be completely lost in *Soesiladeepakius*. The loss of the median apophyses could, then, be a synapomorphy joining this genus with *Lapsamita*. However, to avoid mistakes in the matrix, I am coding “articulated” vs. “fused” apophyses, rather than “present” vs. “absent”.

Likewise, *L. maddisoni* does not help much recover the evolution of the conductor within lapsiines. The reduced projections near the embolus base of *L. estebanensis* and *T. parocula* (conductor-like structure accompanying the embolus; char. 21 in the previous study [6]) are yet to be corfirmed homologous with true conductors. Nevertheless, since true, developed conductors are found in Spartaeinae (such as in *Holcolaetis* Simon and *Allococalodes* Wanless), the possible reduction seen in *Lapsias* and *Thrandina* cannot be considered homologous with that of *Portia*, because this event happened independently within each group. True conductors are rare in salticids, even in non-salticoids. *Lapsamita maddisoni* has a small tegular projection accompanying the embolus (Figs. 2A–C), but this is completely separate from the embolus by a flexible area and its homology with conductor-like structures of *Lapsias* and *Thrandina* is not certain. It is more likely that this structure represents a new, incomparable projection arising at the distal end of the tegulum of *L. maddisoni*. Therefore, this character was coded as “?” for the new species, as in *S. arthrostylus*.

**Figure 4:**
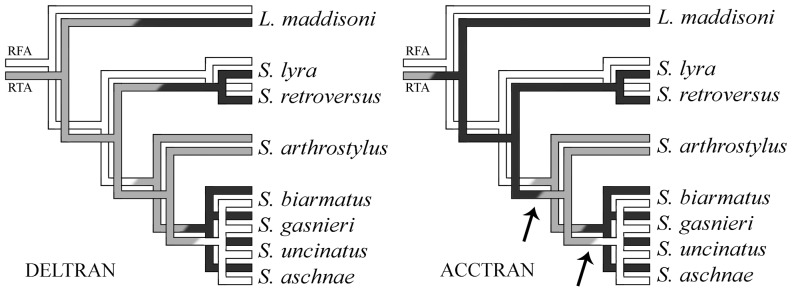
Comparison of optimizations of changes in the retrolateral femoral apophysis (RFA, upper tree) and retrolateral tibial apophysis (RTA, lower tree) in male palps of species of genera *Lapsamita* and *Soesiladeepakius*. White branches represent absence of apophyses (or poorly developed apophyses), gray branches represent moderately developed apophyses and black branches represent elongate or well developed apophyses. RFA shows a straight forward evolution but changes in RTA are ambiguous. Left tree shows a delayed transformation (DELTRAN) of the RTA, becoming independently well developed in *L. maddisoni* and *S. lyra* + *S. retroversus* and poorly developed in the 4-taxon polytomy. Right tree shows an accelerated transformation (ACCTRAN) of the RTA, first becoming elongate at the base of the tree, reducing to moderately developed and then to poorly developed within *Soesiladeepakius*. The two arrows indicate coincidence in changes of RFA and RTA along the tree under ACCTRAN interpretation.

Another character in this new data set whose evolution renders difficult interpretation concerns the length of the RTA (char. 13). The previous study [6] found that the moderately long RTA, reaching only the proximal tegulum (state 0) was plesiomorphic for lapsiines, being present in *P. africana*, *T. parocula*, *G. bryicola*, *G. sacha* and *S. arthrostylus*. The reduced, almost inconspicuous RTA (state 2) appeared joining some species of *Soesiladeepakius* (*S. biarmatus* + *S. gasnieri* + *S. uncinatus* + *S. aschnae*) and homoplastically in *Lapsias estebanensis*. The change to a very long RTA, reaching the distal portion of the tegulum (state 1), was recovered in that study as a synapomorphy of *S. lyra* + *S. retroversus*. The inclusion of *Lapsamita maddisoni* in the analysis, however, has blurred this interpretation. It is clear that state 0 (moderate) is plesiomorphic and that state 2 (reduced RTA) happened independently in *Lapsias* and within *Soesiladeepakius*. The evolution of the elongate RTA (state 1), however, could be reinterpreted with the addition of *L. maddisoni*. If we consider the ambiguity as having a DELTRAN, the long RTA evolved twice, in *S. lyra* + *S. retroversus* and in *L. maddisoni* (Fig. 4, left tree). The ACCTRAN algorithm, on the other hand, suggests that the change from a moderately long RTA (the plesiomorphic form) to an elongate RTA could also be a synapomorphy joining *Soesiladeepakius* and *L. maddisoni* (Fig. 4, right tree). Moreover, that the RTA reduction occurring within *Soesiladeepakius* would have had two steps: first, back to the moderately long RTA (still seen in *S. arthrostylus*) and then greatly reduced in the remaining species of the genus.

The ACCTRAN optimization of RTA length found in this study highlights the biology and sexual behavior in *Soesiladeepakius*. Males of salticids, in general, as in other members of the RTA-clade [15], use the RTA to anchor their palps onto a diversity of pockets and other structures present on the epigynal plate of females before they initiate sperm transfer. Once the palpal tibia is fixed to the female abdomen, the male pumps blood into the hematodocha that attaches the bulb to the cymbium and other hematodochae that hold the bulb sclerites together, causing the complex to expand and the embolus to enter the copulatory opening on the female until it reaches the spermatheca, where the sperm is deposited. Based on palp description of several species of *Soesiladeepakius* [6], in which RTA are strongly reduced or lost, one can imagine that the standard anchoring mechanism is somehow modified within this group.

Despite the fact that we do not know with certainty the functions of femoral apophyses in these spiders, I hypothesize that, with the development of spurs and apophyses on the male palpal femur, the task of anchorage could have been slowly transferred to these new structures. This would have reduced the selective pressure for a developed RTA (by slowly reducing its use/function) and allowed size reduction. Although we barely know the basics of mating anchorage mechanisms in spiders in general to support this hypothesis, the reduction and loss of the elongate RTA are completely coincident with the rise and development of male femoral apophyses within *Soesiladeepakius*, appearing in the same branches of the tree with ACCTRAN interpretation (Fig. 4, arrows in right tree).
